# Programmable Ce6 Delivery via Cyclopamine Based Tumor Microenvironment Modulating Nano-System for Enhanced Photodynamic Therapy in Breast Cancer

**DOI:** 10.3389/fchem.2019.00853

**Published:** 2019-12-05

**Authors:** Chan Feng, Lv Chen, Yonglin Lu, Jie Liu, Shujing Liang, Yun Lin, Yongyong Li, Chunyan Dong

**Affiliations:** ^1^Cancer Center, Shanghai East Hospital, Tongji University, Shanghai, China; ^2^The Institute for Biomedical Engineering & Nano Science (iNANO), School of Medicine, Tongji University, Shanghai, China

**Keywords:** chlorin e6, cyclopamine, photodynamic therapy, drug delivery, breast cancer

## Abstract

Photodynamic therapy (PDT) has shown great promise in breast cancer treatment. However, simplex target ligand modification or stimuli release cannot meet the requirement of effective drug delivery to solid tumor tissue. To overcome continuous bio-barriers existing in the tumor microenvironment, multi-stage response drug delivery was desirable. Herein, we developed a unique tumor microenvironment tailored nanoplatform for chlorin e6 (Ce6) delivery. We chose bovine serum albumin (BSA) as “mother ships” material for effective tumor periphery resident, cyclopamine (CYC) as extracellular matrix (ECM) inhibitor and synergistic anti-tumor agent, and diselenide containing amphiphilic hyaluronic acid-chlorin e6 polymers (HA-SeSe-Ce6) synthesized as “small bombs” for internal tissue destruction. The above three distinct function compositions were integrated into an independent CYC and HA-SeSe-Ce6 co-delivery albumin nano-system (ABN@HA-SeSe-Ce6/CYC). The obtained nano-system presents good biocompatible, long circulation and effective tumor accumulation. After entering tumor microenvironment, CYC gradually releases to disrupt the ECM barrier to open the way for further penetration of HA-SeSe-Ce6. Subsequently, targeted tumor cell internalization and intracellular redox response release of Ce6 would achieve. Moreover, CYC could also make up the deficiency of Ce6 in hypoxia area, owing to its anti-tumor effect. Improved therapeutic efficacy was verified in a breast cancer cell line and tumor-bearing mice model.

## Introduction

Among various emerging therapies, photodynamic therapy (PDT) is a promising non-invasive therapeutic method for superficial tumors, such as breast cancer (Agostinis et al., 2011; Wang D. et al., 2018). One of the most widely used photosensitizers is chlorin e6 (Ce6) (Du et al., 2016; Feng et al., 2019). However, the poor water solubility of photosensitizers hinders their clinical application. In addition, off-target activation of photosensitizers leads to serious side effects (Liu et al., 2017). Therefore, improved delivery of hydrophobic photosensitizers leveraging nanoscale system is desirable. In the past decades, although the various sophisticated chemical design and multi-functional nanoscale systems have developed, cancer nanomedicine still facing challenges for enhancing clinical benefits. Just target ligand modified and stimuli release no longer meet the requirement of effective drug delivery to solid tumor tissue. Despite nanoscale substances preferentially accumulate in tumor tissue than in normal tissue due to permeability and retention effect (EPR effect), abnormal tumor microenvironment with heterogeneous structure often leads to the perivascular area and tumor periphery resident of nanoparticles (Overchuk and Zheng, 2018). Therefore, the design of tumor microenvironment tailored multi-stage photosensitizers delivery is essential.

Due to the lack of mature vessels inside the tumor, it is unable to maintain adequate perfusion of internal tumor tissues (Niu et al., 2018). In addition, tightly packed tumor cells, dense extracellular matrix (ECM) and high interstitial fluid pressure, leading to growth-induced stress, act as biological barriers that further restrict nanoparticle infiltration into the tumor parenchyma after extravasation from vessels (Yang and Gao, 2017; Wang S. et al., 2018). For this dilemma, one of the promising strategies is reducing ECM to open the way for nanoparticles penetration. Cancer-associated fibroblasts (CAFs) play an indispensable role in the formation of ECM, owing to abnormal activation of Hedgehog (Hh) signaling pathway. This pathway is initiated by binding tumor cell-derived Hh ligands to patched 1 (Ptch 1) receptor in CAFs membrane, subsequently, the inhibition of smoothened (SMO) protein is relieved, which triggers activation of the glioma-associated oncogene transcription factor (Gli1-2), leading to downstream genes expression and abundant ECM production (Zhang et al., 2018). Cyclopamine (CYC), a kind of hydrophobic steroid alkaloid, can target the SMO receptor on cancer cells and CAFs to inhibit Hh signaling pathway (Che et al., 2013; Feng et al., 2018). Therefore, CYC is a superexcellent candidate to disrupt the ECM barrier in the tumor microenvironment, as well as an effective anticancer agent.

**Scheme 1:**
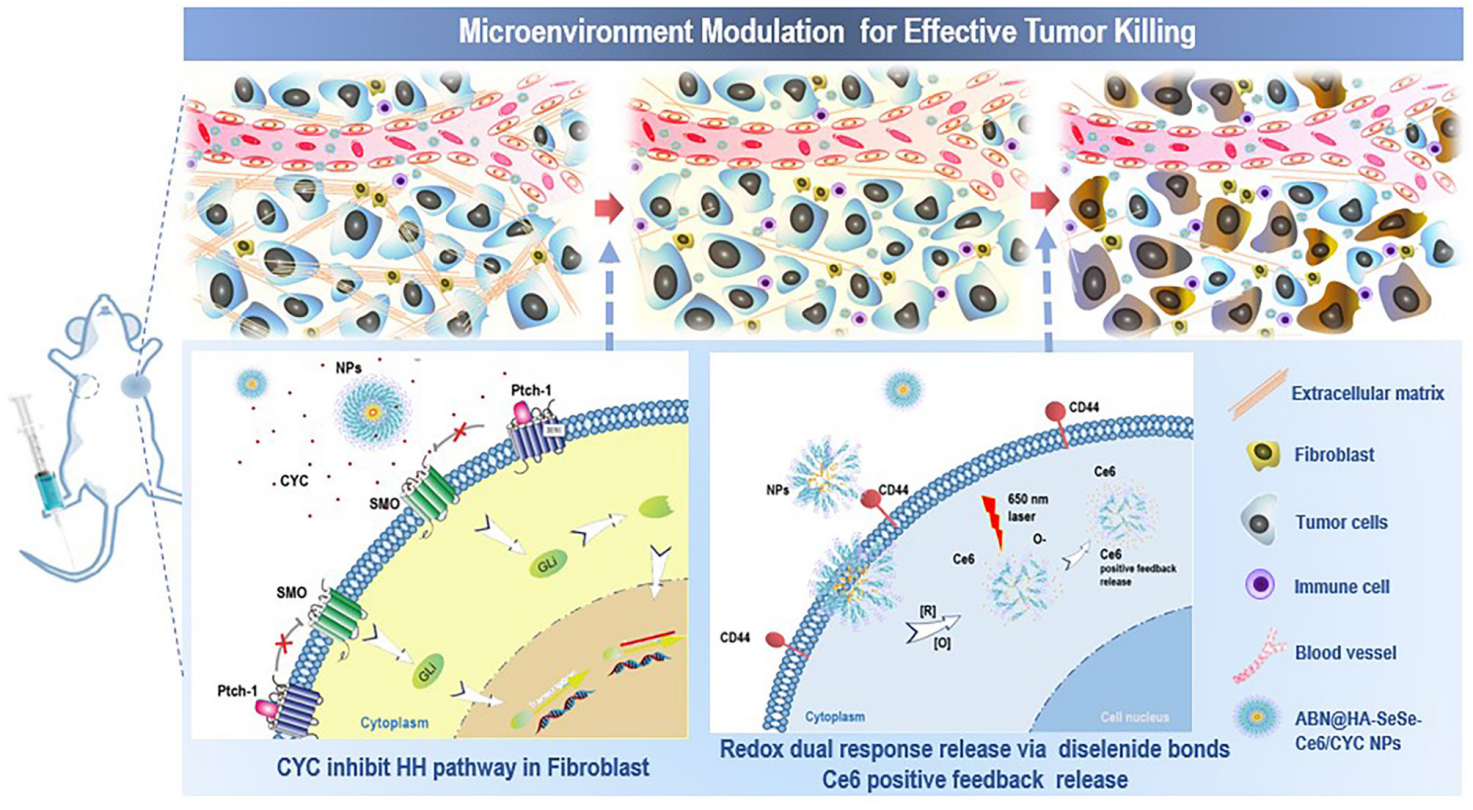
Schematic illustration of tumor microenvironment modulation effect of ABN@HA-SeSe-Ce6/CYC NPs for enhanced PDT. After intravenous injection, NPs will accumulate in tumor periphery and release CYC to disrupt ECM via inhibiting HH pathway in CAFs. Due to the alleviation of solid stress, gradually dissociated ABN@HA-SeSe-Ce6/CYC NPs and smaller HA-SeSe-Ce6 molecules released from the main body will further infiltrate to deeper tumor tissue. Then after targeted tumor cell internalization via recognization of CD44+with HA, diselenide linkages of HA-SeSe-Ce6 molecules will further cleavage to release Ce6 in intracellular redox condition or singlet oxygen (^1^O_2_) existence. In addition, dead tumor cells lead to more immune cells infiltration.

After collapsing the ECM barrier, further diffusion to deeper tumor sites and tumor intracellular smart drug release would be the other two indispensable drug delivery stage. For the former, it was reported that “Cluster Bomb” design could benefit for high-performance tumor suppression (Lei et al., 2017). That is, smaller components, which fall off from the nanoparticles stranded in the tumor periphery, will be easier to infiltrate to deeper tumor tissue. As for the latter stage, these smaller “bomb” components would be better to modify tumor target ligands for effective tumor cells internalization and equip with sensitive linkages for tumor intracellular triggered drug release. Hyaluronic acid (HA), a natural anionic hydrophilic polysaccharide targeting differentiation 44 (CD44) on the cancer cell membrane, is a promising target ligand candidate due to its desirable biocompatibility, biodegradability, non-immunogenicity, and easy functionalization (Choi et al., 2011; Xia et al., 2018a; Feng et al., 2019). In addition, the diselenide bond has particular advantages owing to its high sensitivity to redox condition and singlet oxygen (^1^O_2_), which makes PDT positive drug release theoretically possible (Xia et al., 2016, 2018b; Sun et al., 2017). Herein, a tumor microenvironment tailored multi-stage delivery system based on CYC loaded albumin nanoformulation and was designed for improved PDT therapy in breast cancer treatment (Scheme 1). In our previous study, CYC loaded bovine serum albumin (BSA) nanoparticles showed effective and enduring tumor tissue accumulation and extracellular retention which increase binding of CYC and SMO membrane receptors (Feng et al., 2018). Therefore, we chose BSA as “mother ships” material to load CYC and HA-SeSe-Ce6 via heat-mediate assembling to form co-delivery albumin nanosystem (ABN@HA-SeSe-Ce6/CYC). When nanosystem accumulates in the tumor, CYC will release to inhibit ECM barriers and exert a synergetic anti-cancer effect. Moreover, HA-SeSe-Ce6 as smaller “bomb” component will release and further infiltrate to deeper tumor tissue. In the redox condition of tumor intracellular or existence of ^1^O_2_, diselenide bonds would cleave and lead to Ce6 release (Feng et al., 2019). The improved PDT anticancer effects via tumor microenvironment tailored multi-stage delivery was investigated in breast cancer cell line and breast cancer-bearing mice.

## Materials and Methods

### Materials

Chlorin e6 was obtained from J&K Scientific, Ltd. Cyclopamine was obtained from Hitsan Biotechnology Co., Ltd. (Shanghai, China). Bovine serum albumin, 2-(N-morpholino) ethanesulfonic acid (MES), C4H12N2Se2·2HCl, EDC·HCl, and NHS were obtained from Sigma-Aldrich (Shanghai) Trading Co., Ltd. Hyaluronic acid was purchased from Ruixi biotechnology Co., Ltd.

### Synthesis of HA-Sese-Ce6 Polymers

Five milligrams of HA and 2.5 mg C4H12N2Se2·2HCl were dispersed in 5 ml PBS (pH 7.4). Thirty microliters of EDC·HCl (50 mg/ml) and 20 μl NHS were added to above mixture and stirred at room temperature for 4 h. Ten milligrams of Ce6 was dispersed in 500 μl DMSO, then pipetted 225 μl Ce6 solution and added dropwise to above mixture liquid and stirred for another 4 h. The solution was collected and transferred to centrifugal ultrafiltration tube (10 kDa mol. Cutoff, Pall Corporation, USA) and centrifuged at 6,000 rpm for 15 min. free Ce6, HA, C4H12N2Se_2_·2HCl, and PBS were removed by washing three times with HyPure water. Then, HA-sese-Ce6 solution was 10 times concentrated.

### Preparation of ABN@HA-Sese-Ce6/CYC NPs

Two milligrams of SDS was added to the above HA-sese-Ce6 solution and stirred for 1 h. Two milligrams or milliliters of BSA solution was prepared by adding 5 mg BSA powder to 2.5 ml MES buffer (50 mM, pH = 6.0). Two hundred microliters of CYC ethanol solution (10 mg/ml) and 200 μl HA-sese-Ce6 solution (containing Ce6 2 mg) was slowly added to the BSA solution, respectively. Subsequently, the mixture solution was heated in a silicone oil bath at 70°C and stirred at 800 rpm for 50 s. and the assembly process was stopped by immersing in ice water immediately. The solution was transferred to 100 kda mol. Cutoff centrifugal ultrafiltration tube and centrifuged at 4,500 rpm at 25°C for 20 min. It was washed trice with HyPure water to remove MES, free CYC, and free Ce6.

### Characterization of Nanoparticles

The morphology of nanoparticles was measured by high-resolution transmission electron microscopy (FEI Tccnai G2 F20 S-Twin). Twenty microliters solution of nanoparticles (2 mg/ml) was dripped on carbon-coated copper grid, after 1 min, excess liquid was removed by filter paper. Then add 10 μl 1% uranyl acetate for another 30 s. The TEM samples were dried in the shade at room temperature. The size of nanoparticles was detected by Nano-ZS 90 Nanosizer (Malvern Instruments, UK). Ultraviolet-visible (UV) spectra was recorded by UV spectrophotometer (Varian).

### *In vitro* Drug Release Behavior of ABN@HA-Sese-Ce6/CYC NPs

Two milliliters solution of nanoparticles (containing 5 mg CYC) in dialysis tube was immerged in 50 ml PBS buffer. At predetermined time points, 1 ml of the buffer solution was taken out to measure CYC concentration, and then add 1 ml fresh PBS buffer to keep the volume of solution. The CYC content was measured by LC-MS (the mobile phase: acetonitrile: 0.1% formic acid 80:20 solution, flow rate 0.35 ml/min). Ten milliliters solution of nanoparticles (containing 6 mg Ce6) was individually transferred to 5 dialysis tubes, and then immerged in 50 ml PBS, 10 mM GSH, 100 mM GSH, 1 mM H_2_O_2_, 10 mM H_2_O_2_ solution, respectively. At predetermined time points, 1 ml buffer solution was taken out to measure Ce6 concentration via UV spectrophotometer at 404 nm. Two milliliters solution of nanoparticles (containing 1 mg Ce6) was exposed to 650 nm (20 mW/cm^2^) for 10 min, then immerged in PBS solution to measure Ce6 concentration at different time points.

### Singlet Oxygen (^1^O_2_) Measurement

Take out the buffer solution from groups treated with 10 mM GSH and 10 mM H_2_O_2_ described in method 2.5, and add ^1^O_2_ detecting reagent SOSG to the solution (final concentration: 1 μM). Then, the above solutions were exposed to 650 nm laser for 90, 180, 270, 360, and 450 s. The fluorescence was measured by Hitachi F2500 luminescence spectrometer (emission spectra: 490–700 nm, excitation wavelength: 488 nm).

### *In vitro* Cytotoxicity Test

In 37°C incubator with 5% carbon dioxide, the mouse breast cancer cell line 4T1 cells were cultured in DMEM medium containing 10% FBS. 1 × 10^4^ cells/well 4T1 cells were seeded in 96 wells plate and incubated for 12 h. Then add free Ce6, ABN@HA-sese-Ce6 and ABN@HA-sese-Ce6/CYC solutions containing different Ce6 concentrations (0.25, 0.5, 1.2 μM), respectively. The same volume of serum-free DMEM was added as control treatment. The half of above cells were exposed to 650 nm laser (20 mW/cm^2^) for 5 min, while the other cells were still cultured in the dark. Twenty-four hours later, 10 μl CCK-8 solution was added to every well. After 3 h incubation, the absorbance of each well was detected by the plate reader at 450 nm.

### Targeted Cellular Uptake Assay

1 × 10^5^ cells/ well 4T1 cells were seeded in confocal dishes. After 24 incubation, cells were treated individually with free Ce6 and ABN@HA-sese-Ce6/CYC for 2, 6, and 10 h. Remove the medium of each well and wash with PBS for three times. Then, cells were stained with DAPI for 15 min and washed with PBS. The prepared samples were detected by confocal laser scanning microscope (Leica TCS SP5II, Germany).

### Tumor Models

The animal experiments in this study were carried out according to Tongji University Animal Ethics Guidelines. The animal experiment protocol was approved by Tongji University Animal Ethics Committee.

Five-week-old female BALA/c mice were injected with 5 × 10^5^ 4T1 cells (subcutaneous route). When tumor tissue reached ~500 mm^3^, tumor tissues were excised and cut into 1 mm^3^ tissue blocks. The tissue block was planted in the left mammary fat pad of 5-week-old female BALA/c mice. When the tumor growth large enough, tumor models were further treated.

### *In vivo* Fluorescence Imaging

The breast cancer bearing mice were pretreated with tail vein injection of ABN@CYC (CYC 20 mg/kg) for three time every 2 days. Then the pretreated mice were injected with ABN@HA-sese-Ce6/CYC NPs. At the same time, the mice without pretreatment were inject free Ce6 and ABN@HA-sese-Ce6 (equivalent Ce6 concentration) as control. At predetermined time points, *in vivo* fluorescence imaging was carried out by a Night OWL LB 983 *in vivo* imaging system.

### Photodynamic Therapy in Animals

Plant tumor tissue block in the left mammary fat pad of 5-week-old female BALA/c mice to build animal model. After 7 days, mice were treated individually with PBS, free CYC, free Ce6, ABN@HA-sese-Ce6 and ABN@HA-sese-Ce6/CYC NPs for 7 times (CYC 20 mg/kg, Ce6 2.5 mg/kg, every 2 days). Began with third injection, the mice were exposed to a 650 nm laser (20 mW/cm^2^) for 30 min under anesthesia after each injection. The survival time of five groups of mice was recorded. Mice with tumors exceeding ethical requirements (>2 cm) were euthanized (equivalent to endpoint of observation), and the animals were euthanized using carbon dioxide asphyxia. Excised tumor weight of every group was also measured. Tumor volume and mice body weight were recorded every 2 days, the calculation formula was as following:

Tumor volume = (Length × Width × Width)/2

The major organs (heart, liver, spleen, lung, and kidney) and tumors were excised from mice of different groups. The collected tissues were immerged in 4% paraformaldehyde overnight. Then the tissues were dehydrated in graded ethanol solution and embedded in paraffin. The paraffin sections were prepared for hematoxylin and eosin (HE) and terminal deoxynucleotidyl transferase dUTP nick end labeling (TUNEL) staining. In addition, tumor microenvironment fibronectin and immune cells distribution were performed by immunofluorescence through tumor tissue frozen section, and detected by confocal laser imaging microscope.

### Statistical Analysis

All values of this study are presented as mean ± SD. The statistical significance of the data was determined by one-way single factorial analysis of variance (ANOVA). Significant differences are expressed as ^*^p ≤ 0.05, ^**^ ≤ 0.01, ^***^ ≤ 0.001.

## Results and Discussion

### Nanoparticles Synthetic Strategy and Characterization

**Scheme 2:**
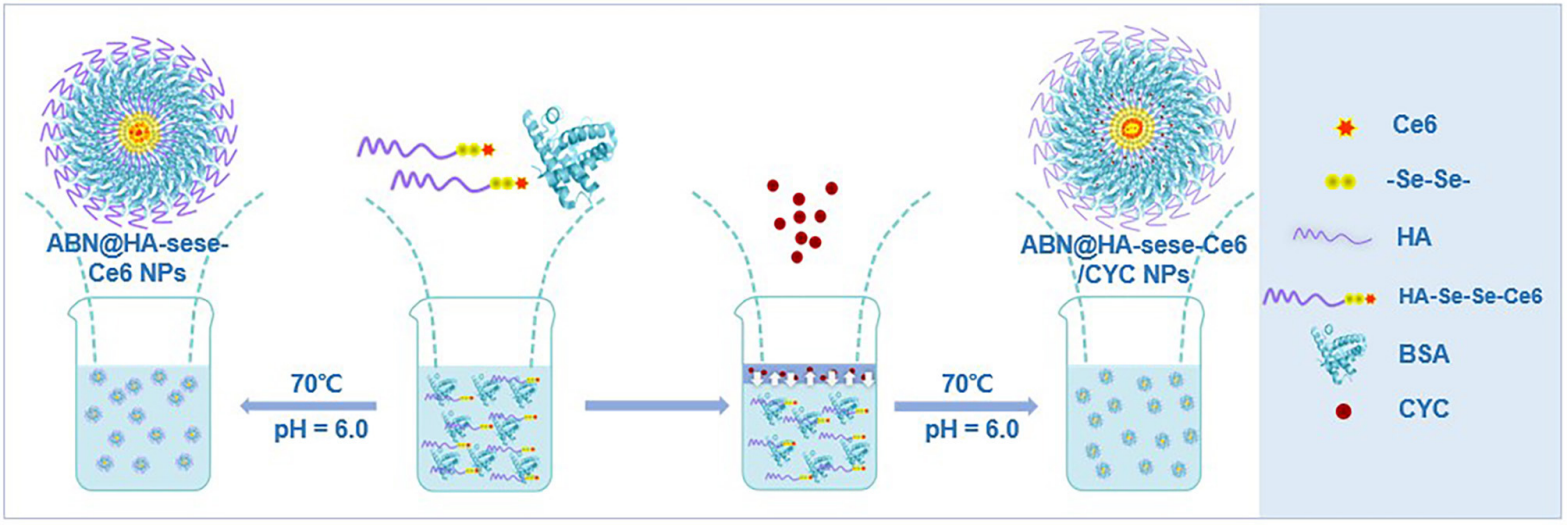
The schematic process of ABN@HA-sese-Ce6/CYC NPs and ABN@HA-sese-Ce6 NPs preparation. HA-sese-Ce6 molecules and BSA were added to MES solution and mix well. Then slowly add CYC ethanol solution to the mixture to form temporary binary solution. Subsequently, the hydrophobic sites on BSA was exposed at 70°C, which facilitate the process of assembling with CYC and HA-sese-Ce6 and form compact ABN@HA-sese-Ce6/CYC NPs. Besides, ABN@HA-sese-Ce6 NPs can be prepared in same way via heating HA-sese-Ce6 molecules and BSA mixture solution for 50 s.

**Figure 1:**
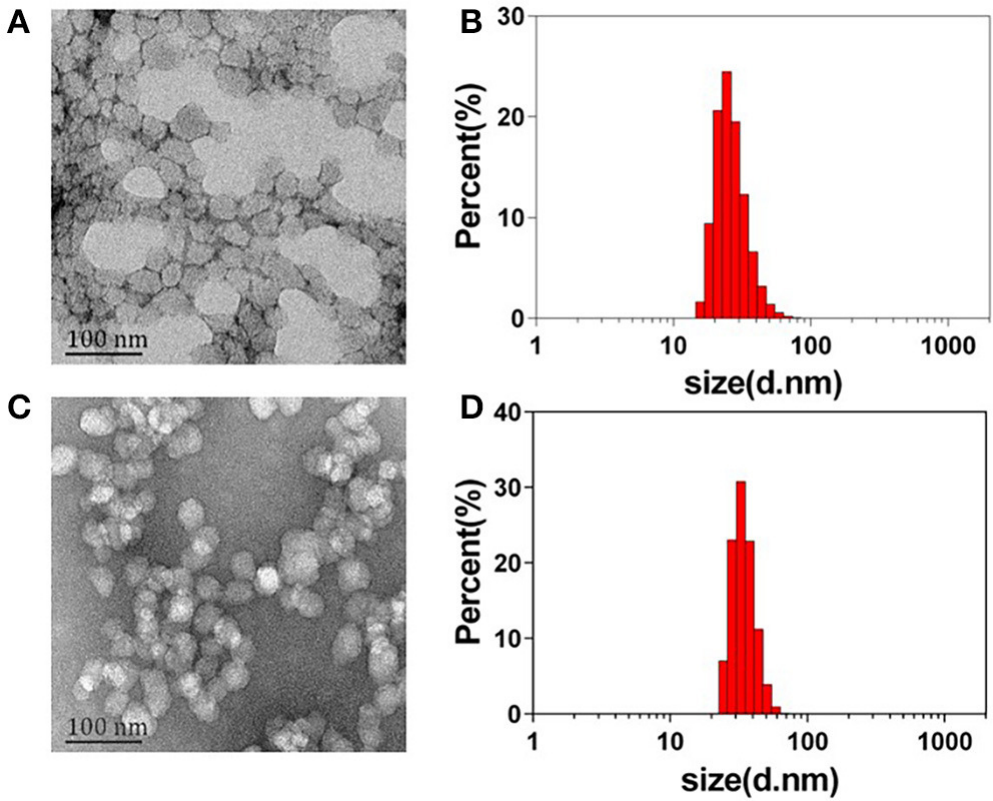
The morphology and size distribution of nanoparticles. **(A)** The TEM image of ABN@HA-sese-Ce6 NPs; **(B)** Hydrate particle size of ABN@HA-sese-Ce6 NPs; **(C)** TEM image of ABN@HA-sese-Ce6/CYC NPs; **(D)** Hydrate particle size of ABN@HA-sese-Ce6/CYC NPs.

The ABN@HA-sese-Ce6/CYC delivery system is consist of three functional parts. Firstly, BSA was chosen as main drug carrier material to integrate every part via transient heat triggered assembling. Then, hydrophobic small molecule CYC acted as tumor microenvironment modulator. At last, we assign the diselenide containing amphiphilic hyaluronic acid-chlorin e6 polymers (HA-SeSe-Ce6) as redox response “small bomb” of delivery system. To avoid its self-assembling, we pretreated HA-SeSe-Ce6 with some SDS to make it stretch. The assembling process is driven by the BSA hydrophobic sites exposure and hydrophobic nucleation effects of hydrophobic agents (Ce6 and CYC). The hydrophobic interaction finally promotes to the formation of ABN@HA-sese-Ce6/CYC with compact assembly. The strategy was shown in Scheme 2. The ABN@HA-sese-Ce6 was prepared as control. The morphology of ABN@HA-sese-Ce6/CYC NPs and ABN@HA-sese-Ce6 NPs were measured by transmission electron microscopy (TEM). As shown in Figure 1, nanoparticles are a spherical shape with a narrow distribution. The average size of ABN@HA-sese-Ce6/CYC NPs was 35 nm, slightly larger than ABN@HA-sese-Ce6 NPs (28 nm).

### Drug Release Behavior of ABN@HA-Sese-Ce6/CYC NPs *in vitro*

**Figure 2:**
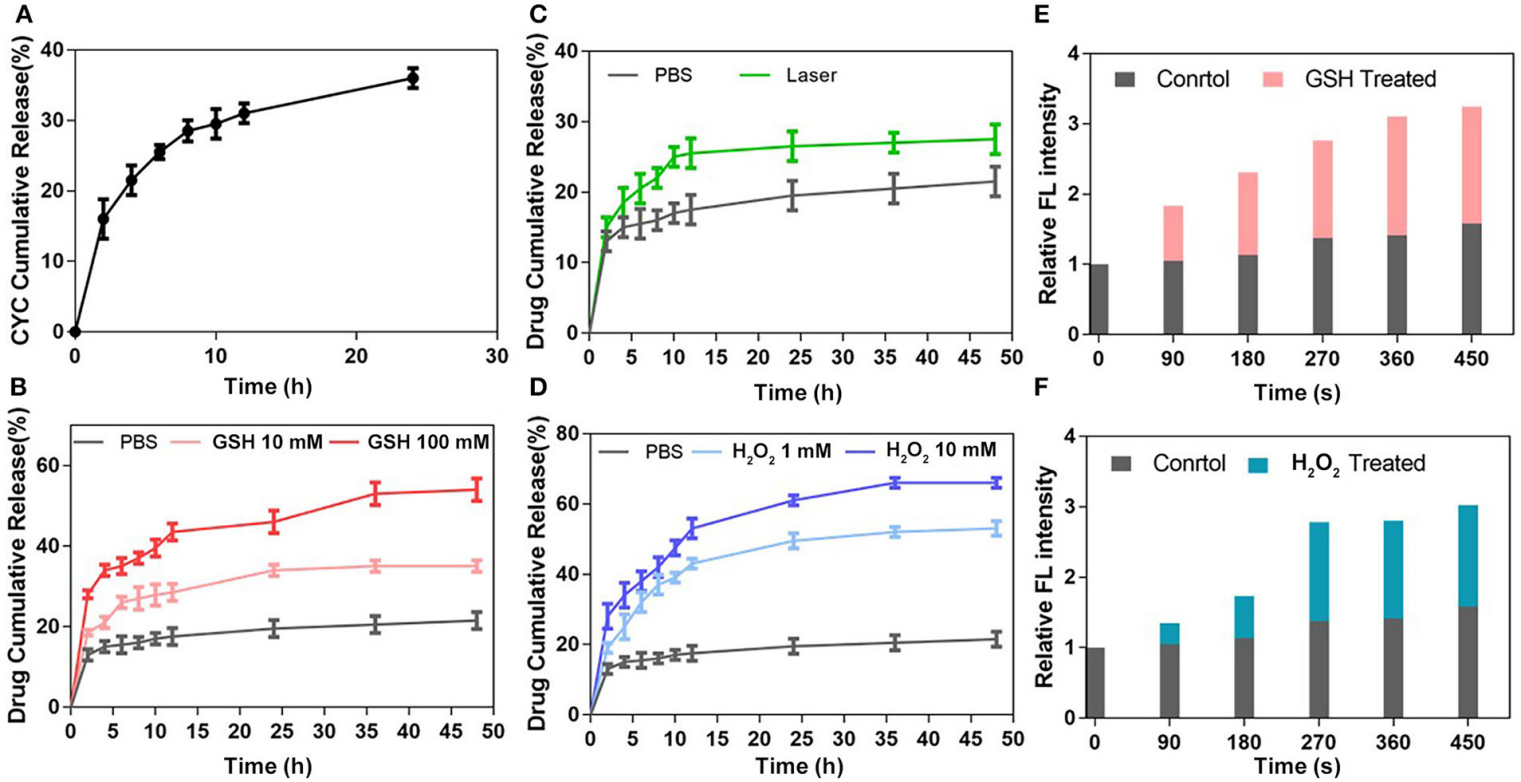
Drug release behavior of ABN@HA-sese-Ce6/CYC NPs. **(A)** The Ce6 release of ABN@HA-sese-Ce6/CYC NPs in PBS, 10 mM GSH, 100 Mm GSH, respectively. **(B)** The Ce6 release of ABN@HA-sese-Ce6/CYC NPs in PBS, 1 mM H_2_O_2_, 10 mM H_2_O_2_. **(C)** The Ce6 release of ABN@HA-sese-Ce6/CYC NPs when treated with 650 nm laser (20 mW/cm^2^). Without the laser treatment group as a control. **(D)** The CYC release of ABN@HA-sese-Ce6/CYC NPs in PBS. **(E)** Singlet oxygen generated by the released Ce6 of ABN@HA-sese-Ce6/CYC NPs in 10 mM GSH treated group when exposed to 650 nm laser (20 mW/cm^2^). PBS group as a control. **(F)** Singlet oxygen generated by the released Ce6 of ABN@HA-sese-Ce6/CYC NPs in 1 mM H_2_O_2_ treated group when exposed to 650 nm laser (20 mW/cm^2^). PBS group as a control.

The CYC release behavior was measured in PBS (pH 7.4) at 37°C. As shown in Figure 2A, the CYC cumulative release ratio increased in the first 8 h and reached about 30%, then slowed down. Due to the existence of diselenide bonds between the Ce6 and HA, it was easier for CYC to release from NPs than Ce6 in PBS, which provided the benefit for CYC to inhibit EMC first. As shown in Figure 2B, there was a small quantity of Ce6 release in PBS solution, which could be explained by mixed free Ce6 physical adsorption. In theory, diselenide bonds will cleavage to promote Ce6 release while nanoparticles exposed in redox conditions. To investigate the redox-sensitive release of Ce6, we measured Ce6 release behavior in GSH and H_2_O_2_ solutions with different concentration at 37°C. As shown in Figure 2B, Ce6 cumulative release presented GSH concentration-dependent. In 100 mM GSH group, the higher plateau of Ce6 cumulative release (44%) was observed in 12 h, while Ce6 cumulative release only reached 28% in 12 h in 10 mM GSH group. Overall, GSH treated group showed the obviously increased release of Ce6, compared with the PBS group.

Similar to GSH treated group, Ce6 cumulative release also presented H_2_O_2_ concentration-dependent increase. The results of H_2_O_2_ treated group were shown in Figure 2D, in 10 mM H_2_O_2_ group, Ce6 cumulative release reached a plateau at 55% in 12 h, while 1 mM H_2_O_2_ group reached a plateau at 43% in 12 h. In addition, it was found that more Ce6 cumulative release treated with H_2_O_2_ than GSH, suggesting more sensitivity of diselenide in oxidizing condition. ^1^O_2_ would be generated from Ce6 under 650 nm laser, which theoretically breaks part of diselenide bonds to further promote Ce6 release. Therefore, we also investigate the sensitivity of NPs when they exposed to the laser. The results in Figure 2C verified it, and we can find that more Ce6 release in 650 nm laser-treated group, compared with the control group. Finally, we further measured ^1^O_2_ generation of released Ce6 in GSH and H_2_O_2_ treated group respectively to indirectly confirm redox triggered Ce6 release (Figures 2E,F).

### *In vitro* Cytotoxicity and Targeted Cellular Uptake Assay

**Figure 3:**
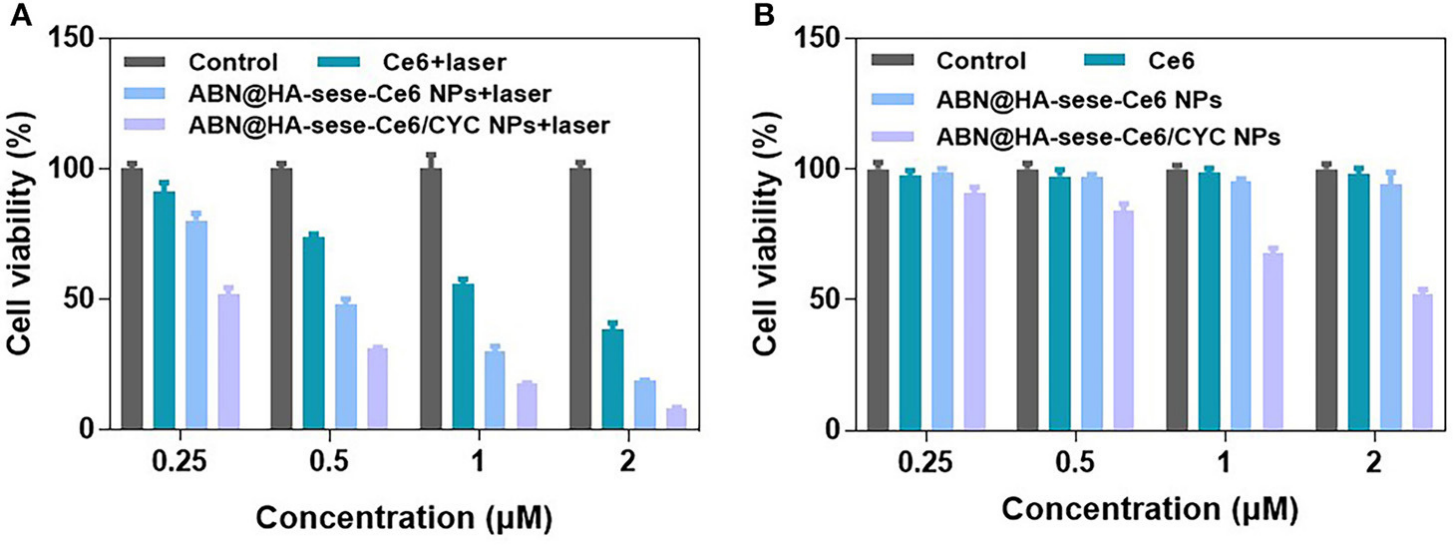
Cytotoxicity assay in 4T1 breast cancer cells. **(A)** Phototoxicity of Ce6, BSA@HA-sese-Ce6, and BSA@HA-sese-Ce6@CYC (equivalent concentration 0.25–2 μM Ce6). Fresh DMEM medium with laser as a control group; **(B)** Dark toxicity of above-treated groups. Fresh DMEM medium as a control group.

We evaluated the *in vitro* phototoxicity and dark cytotoxicity of NPs in mice breast cancer 4T1 cell line. As shown in Figure 3A, ABN@HA-sese-Ce6 NPs had better concentration-dependent phototoxicity than free Ce6, while ABN@HA-sese-Ce6/CYC had the most phototoxicity against 4T1 cell line with <10% cell viability at low Ce6 concentration (2 μM). In the condition without laser exposure, ABN@HA-sese-Ce6 and free ce6 exhibited no significant toxicity (Figure 3B). As expected, ABN@HA-sese-Ce6/CYC NPs was effective at all concentrations, the concentration-dependent dark toxicity was owing to CYC (the CYC concentration was 10 times of Ce6 concentration). Therefore, HH pathway inhibitor CYC provided complementation in cancer inhibition.

**Figure 4:**
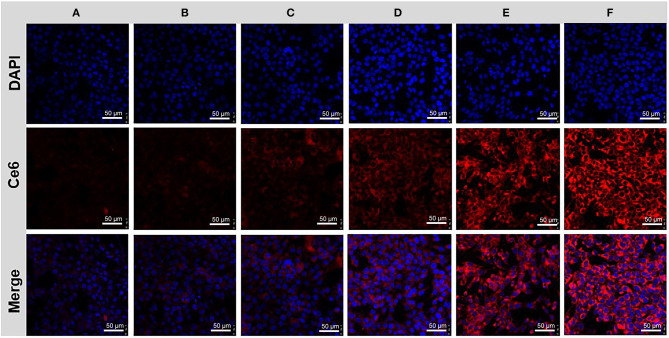
Cancer cell uptake assay. Cells treated with free Ce6 for 2 h **(A)**, 6 h **(C)**, 10 h **(E)**; and BSA@HA-sese-Ce6@CYC for 2 h **(B)**, 6 h **(D)**, 10 h **(F)**.

To further confirm the targeted anti-cancer effect, we investigate the targeting cancer internalization of NPs by CLSM. 4T1 cells were seeded in confocal dishes and treated with free Ce6 and ABN@HA-sese-Ce6/CYC NPs, respectively. After 2, 6, 10 h, cells were stained with DAPI and observed by CLSM. As shown in Figure 4, the cells treated with ABN@HA-sese-Ce6/CYC NPs exhibited stronger fluorescence than free Ce6 treated group in every time point, especially in 6 h (Figures 4C,D). These results verified the targeting effect of HA in CD44+ overexpressed 4T1 cell line.

### *In vivo* Tumor Accumulation of ABN@HA-Sese-Ce6/CYC NPs

**Figure 5:**
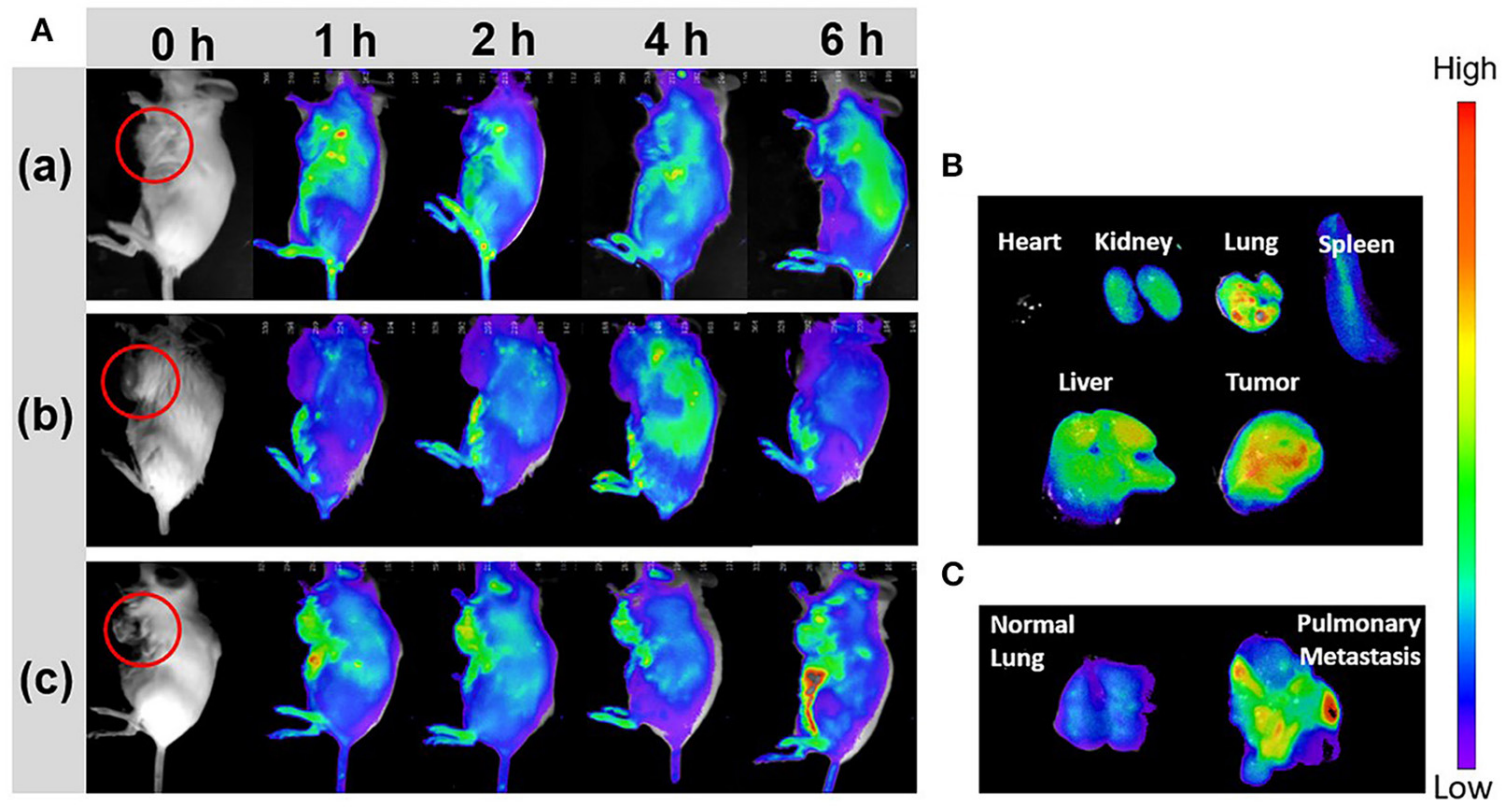
*In vivo* fluorescence imaging. **(A)** 4T1 breast cancer bearing mice were imaged in 1, 2, 4, 6 h after i.v. injection with free Ce6 (a), ABN@Ce6 NPs (b), and ABN@HA-sese-Ce6/CYC NPs (c). **(B)** Florescence images of excised heart, kidney, lung, spleen, liver, and tumor in ABN@HA-sese-Ce6/CYC NPs treated group. **(C)** Florescence images of normal lung and pulmonary metastasis after injection with ABN@HA-sese-Ce6/CYC NPs.

To investigate the CYC effect for improving tumor accumulation, the breast cancer-bearing mice were pretreated with ABN@CYC for 3 times every 2 days. Then the pretreated mice were injected via tail vein with ABN@HA-sese-Ce6/CYC NPs. At the same time, 4T1 breast cancer-bearing mice without pretreatment were injected with free Ce6 and ABN@HA-sese-Ce6 (equivalent Ce6 concentration) as control. At 1, 2, 4, 6 h after injection, *in vivo* fluorescence imaging was performed. In free Ce6 group, fluorescence signal was observed in part of tumor in fist 1 h, meanwhile, the lung, liver, kidney, and bladder accumulations were also obvious (Figure 5Aa). After 1 h, the fluorescence in tumor tissue was weakened and others organ accumulation was enhanced. In ABN@HA-sese-Ce6 group, there was no obvious tumor accumulation until 4 h, and the fluorescence was just limited to peripheral area of tumor (Figure 5Ab). As shown in Figure 5Ac, the most strong and enduring tumor fluorescence signal was exhibited in ABN@HA-sese-ce6/CYC NPs treatment group, compared with ABN@HA-sese-Ce6 and free Ce6 group without pretreatment. The results confirmed the significant effect of CYC for enhancing tumor accumulation. Moreover, we also excised heart, kidney, lung, spleen, liver, and tumor from the mouse at 6 h in ABN@HA-sese-ce6/CYC NPs group. As shown in Figure 5B, the strong fluorescence was observed in tumor and liver, suggesting significant targeted tumor accumulation and liver clearance. Besides, accumulation in pulmonary metastasis was also remarkable. After ABN@HA-sese-ce6/CYC treatment, it was found that stronger fluorescence accumulation in pulmonary metastasis than normal lung (Figure 5C).

### *In vivo* Anti-tumor Effects and ECM Modulation Evaluation

**Figure 6:**
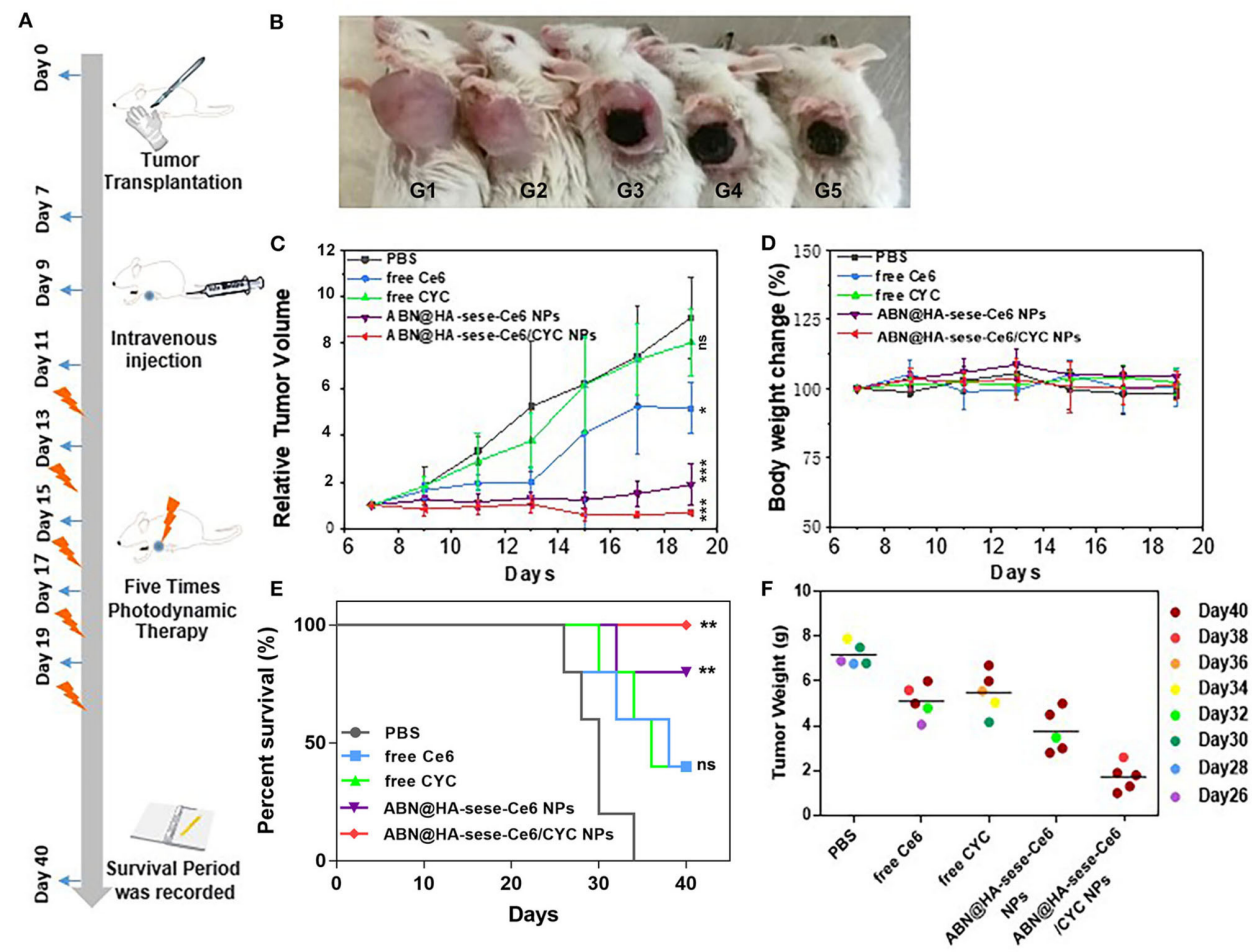
Anti-tumor effects in breast cancer bearing mice model. **(A)** Schematic illustration of animal experiments. **(B)** Photos of tumor bearing mice in different treatment group (from left to right: PBS, free CYC, free Ce6, ABN@HA-sese-Ce6 NPs, ABN@HA-sese-Ce6/CYC NPs treatment group). **(C)** Tumor volume curve of mice treated with PBS, free Ce6, free CYC, ABN@HA-sese-Ce6 NPs, ABN@HA-sese-Ce6/CYC NPs (*n* = 5). The data are mean ± SD, ^*^p < 0.05, ^**^ < 0.01, ^***^ < 0.001 vs. PBS group. **(D)** Body weight change curve of mice in above groups. **(E)** The survival curve of mice in different groups. ^*^p < 0.05, ^**^ < 0.01, ^***^ < 0.001 vs. PBS group (*n* = 5). **(F)** Excised tumor weight of mice in above treatment groups when natural death or on Day 40 (endpoint).

*In vivo* anti-cancer therapeutic experiment was carried on in the 4T1 orthotopic mammary fat pad tumor bearing mice. As schematic illustration shown in Figure 6A, we termed the day when we planted the tumor blocks in mice as Day 0. From Day 7 to Day 19, tumor bearing mice were treated with PBS, free CYC, free Ce6, ABN@HA-sese-Ce6, and ABN@HA-sese-Ce6/CYC NPs for 7 times, respectively. From Day 11 to Day 19, mice were exposed to 650 nm laser for 30 min after every injection (20 mW/cm^2^, at 1 h after injection via tail vein).

As shown in Figures 6B,C, there was a remarkable difference between different treated groups. Compared with PBS control group, free CYC group exhibited minimal anti-tumor effect, free Ce6 showed much more remarkable anti-tumor effect. By contrast, ABN@HA-sese-Ce6 and ABN@HA-sese-Ce6/CYC NPs had strong anti-tumor effect, especially ABN@HA-sese-Ce6/CYC NPs reduced the tumor volume obviously. Besides, remarkable necrosis and escharosis were observed in three Ce6 treated groups (Figure 6B). Consistent with tumor volume change curve, the tumor weight of ABN@HA-sese-Ce6/CYC NPs treated group were also lightest among all groups (Figure 6F). Moreover, survival period of mice was recorded until Day 40, and the mice of ABN@HA-sese-Ce6/CYC NPs were all survival (Figure 6E). During the process of therapy, there was no significant body weight change of mice in every treatment group (Figure 6D).

**Figure 7:**
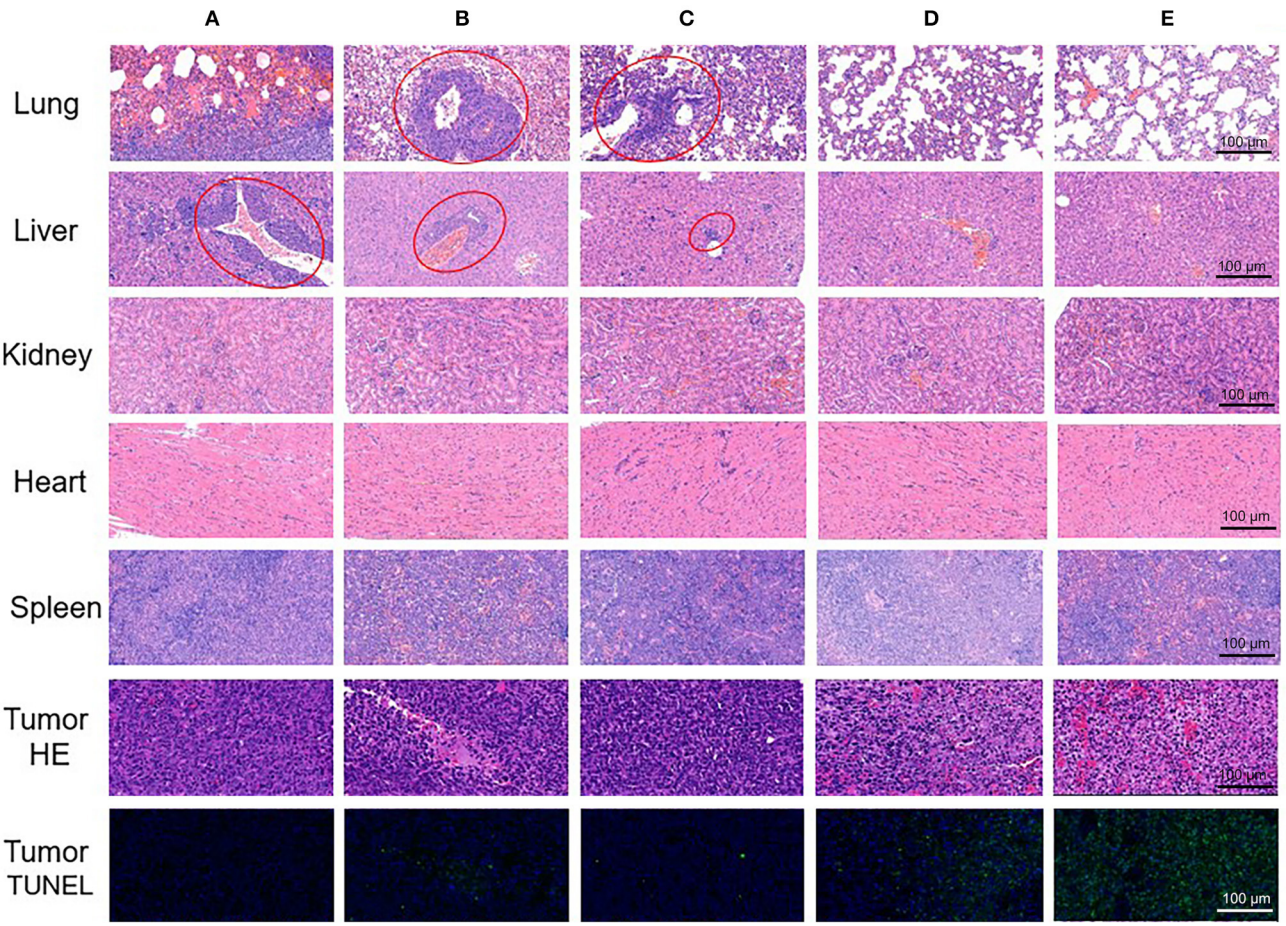
The pathological slices of excised tumor and organs (lung, liver, kidney, heart, spleen) in control group **(A)**, free Ce6 group **(B)**, free CYC group **(C)**, ABN@HA-sese-Ce6 NPs group **(D)**, and ABN@HA-sese-Ce6/CYC NPs group **(E)**. Red circles represent tumor metastasis.

To further confirm anti-tumor and metastasis inhibition effects in histological level, we excised tumors and organs from treated mice, and prepared pathological slices. As TUNEL staining and HE staining of tumors shown in Figure 7, the most numbers of necrotic and apoptosis cells were found in ABN@HA-sese-Ce6/CYC treated group, which was consistent with above animal experiment results. Besides, it's worth noting that obvious liver metastasis and pulmonary consolidation due to tumor metastasis can be observed, except ABN@HA-sese-Ce6 and ABN@HA-sese-Ce6/CYC group. Compared with ABN@HA-sese-Ce6 group with scattered metastasis focuses, there was no significant metastasis found in ABN@HA-sese-Ce6/CYC group, suggesting the good metastasis inhibition effect of ABN@HA-sese-Ce6/CYC NPs.

**Figure 8:**
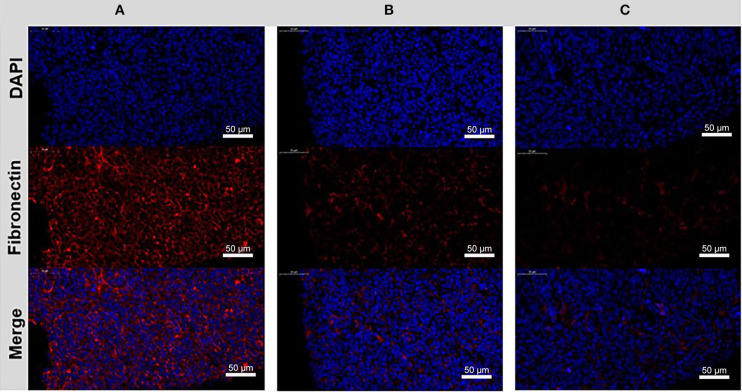
The disrupting effect of CYC treatment to fibronectin in the tumor ECM. Immunofluorescence images of control group **(A)**, free CYC treatment group **(B)**, ABN@HA-sese-Ce6/CYC NPs treatment group **(C)** after 4 times i.v. injection. Blue, nuclei. Red, Fibronectin.

Moreover, the ECM modulation effect was also investigated. We excised tumor tissues from the mice (PBS, free CYC, ABN@HA-sese-Ce6/CYC treated groups), and prepared tissue sections. The Fibronectin of tumor tissue was label by red fluorescence. Compared with PBS control group, weaken red fluorescence was observed in free CYC and ABN@HA-sese-Ce6/CYC treated groups, while the ABN@HA-sese-Ce6/CYC group exhibited the weakest fibronectin fluorescence (Figure 8). It was confirmed that CYC can inhibit the fibronectin expression in tumor tissues, and ABN@HA-sese-Ce6/CYC improve the effects of CYC in some degree.

## Conclusion

In this study, we developed a programmable Ce6 delivery nano-system to promote PDT therapy. Redox response, laser triggered Ce6 release and tumor cells targeted internalization was confirmed *in vitro*. Improved tumor accumulation via EMC inhibition of ABN@HA-sese-Ce6/CYC was confirmed in 4T1 tumor bearing mice. Moreover, enhanced anti-tumor effect, obvious metastasis inhibition as well as extended survival period were observed in animal experiments. Therefore, this Ce6 delivery nano-system with improved tumor targeted delivery via tumor microenvironment modulation, smart drug release and promoted therapeutic efficacy, which provided a promising drug delivery strategy for overcome continuous bio-barriers in anti-tumor delivery.

## Data Availability Statement

All datasets generated for this study are included in the article/supplementary material.

## Ethics Statement

The animal study was reviewed and approved by Tongji University Animal Ethics Guidelines.

## Author Contributions

CF, CD, and YLi designed the experiments. CF, LC, YLu, and JL carried out the experiments. SL and YLin helped analyzing the experimental results. CF wrote the manuscript.

### Conflict of Interest

The authors declare that the research was conducted in the absence of any commercial or financial relationships that could be construed as a potential conflict of interest.
