# Corrigendum: Programmable Ce6 Delivery via Cyclopamine Based Tumor Microenvironment Modulating Nano-System for Enhanced Photodynamic Therapy in Breast Cancer

**DOI:** 10.3389/fchem.2020.608312

**Published:** 2020-11-27

**Authors:** Chan Feng, Lv Chen, Yonglin Lu, Jie Liu, Shujing Liang, Yun Lin, Yongyong Li, Chunyan Dong

**Affiliations:** ^1^Cancer Center, Shanghai East Hospital, Tongji University, Shanghai, China; ^2^The Institute for Biomedical Engineering & Nano Science (iNANO), School of Medicine, Tongji University, Shanghai, China

**Keywords:** chlorin e6, cyclopamine, photodynamic therapy, drug delivery, breast cancer

In the original article, there was a mistake in ^**^Figure 6E^**^ as published. ^**^We made a data processing error of PBS survival curve in Figure 6E^**^. The corrected ^**^Figure 6^**^ appears below.

**Figure 6:**
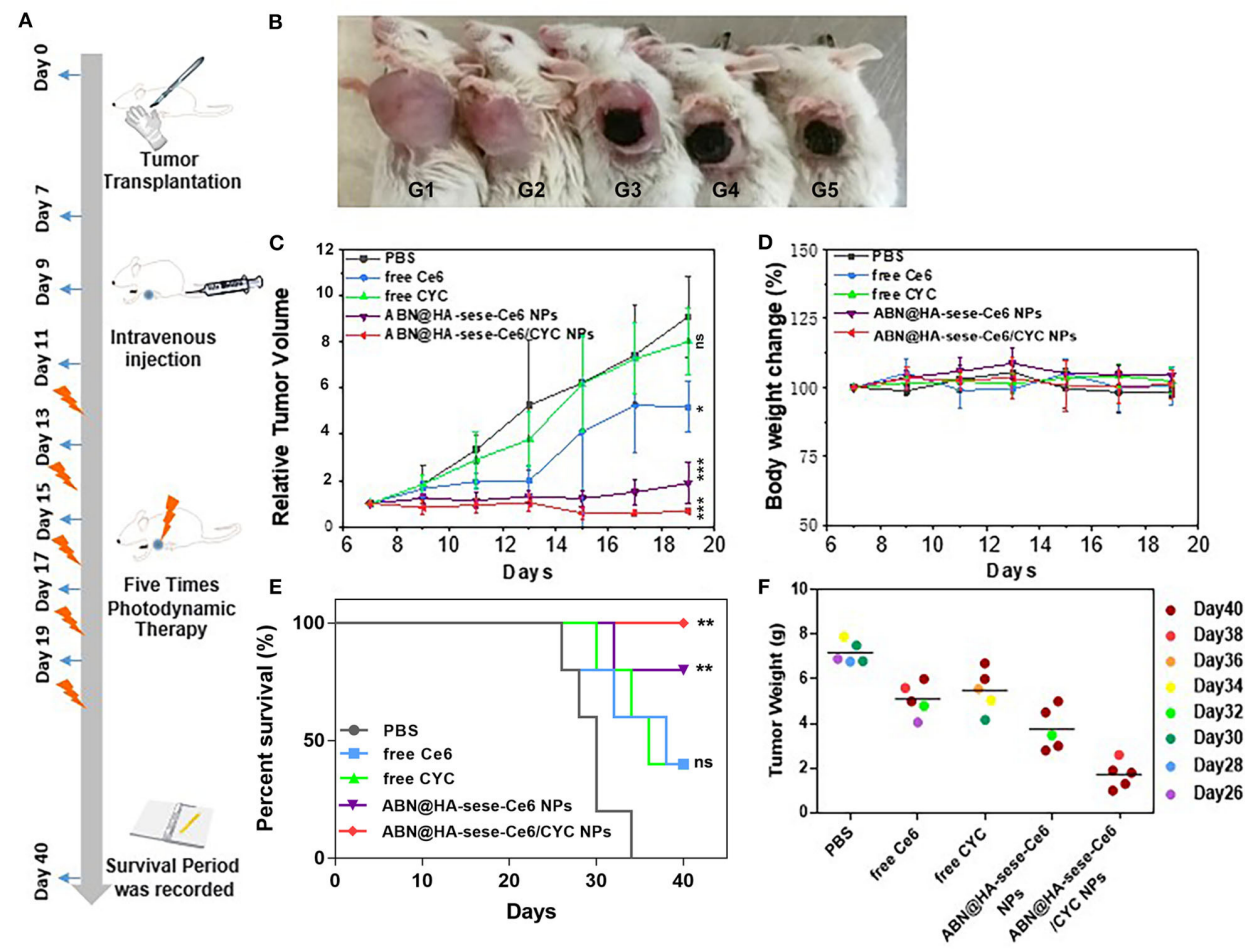
Anti-tumor effects in breast cancer bearing mice model. **(A)** Schematic illustration of animal experiments. **(B)** Photos of tumor bearing mice in different treatment group (from left to right: PBS, free CYC, free Ce6, ABN@HA-sese-Ce6 NPs, ABN@HA-sese-Ce6/CYC NPs treatment group). **(C)** Tumor volume curve of mice treated with PBS, free Ce6, free CYC, ABN@HA-sese-Ce6 NPs, ABN@HA-sese-Ce6/CYC NPs (*n* = 5). The data are mean ± SD, ^*^p <0.05, ^**^ <0.01, ^***^ <0.001 vs. PBS group. **(D)** Body weight change curve of mice in above groups. **(E)** The survival curve of mice in different groups. ^*^p <0.05, ^**^ <0.01, ^***^ <0.001 vs. PBS group (*n* = 5). **(F)** Excised tumor weight of mice in above treatment groups when natural death or on Day 40 (endpoint).

In the original article, there was an error. ^**^There is no detail description of operations involving ethical aspects of experimental animals^**^.

A correction has been made to ^**^Materials and Methods^**^, ^**^Photodynamic Therapy in animals^**^, ^**^Paragraph 1^**^:

^**^Plant tumor tissue block in the left mammary fat pad of 5-week-old female BALA/c mice to build animal model. After 7 days, mice were treated individually with PBS, free CYC, free Ce6, ABN@HA-sese-Ce6 and ABN@HA-sese-Ce6/CYC NPs for 7 times (CYC 20 mg/kg, Ce6 2.5 mg/kg, every 2 days). Began with third injection, the mice were exposed to a 650 nm laser (20 mW/cm^**2**^) for 30 min under anesthesia after each injection. The survival time of five groups of mice was recorded. Mice with tumors exceeding ethical requirements (>2 cm) were euthanized (equivalent to endpoint of observation), and the animals were euthanized using carbon dioxide asphyxia. Excised tumor weight of every group was also measured. Tumor volume and mice body weight were recorded every 2 days, the calculation formula was as following:^**^

The authors apologize for this error and state that this does not change the scientific conclusions of the article in any way. The original article has been updated.

